# Discrimination of Influenza Infection (A/2009 H1N1) from Prior Exposure by Antibody Protein Microarray Analysis

**DOI:** 10.1371/journal.pone.0113021

**Published:** 2014-11-18

**Authors:** Dennis te Beest, Erwin de Bruin, Sandra Imholz, Jacco Wallinga, Peter Teunis, Marion Koopmans, Michiel van Boven

**Affiliations:** 1 Centre for Infectious Disease Control, National Institute for Public Health and the Environment, Bilthoven, The Netherlands; 2 Centre for Health Protection, National Institute for Public Health and the Environment, Bilthoven, The Netherlands; 3 Rollins School of Public Health, Emory University, Atlanta, Georgia, United States of America; 4 Department of Viroscience, Erasmus Medical Centre, Rotterdam, The Netherlands; Alberta Provincial Laboratory for Public Health/University of Alberta, Canada

## Abstract

Reliable discrimination of recent influenza A infection from previous exposure using hemagglutination inhibition (HI) or virus neutralization tests is currently not feasible. This is due to low sensitivity of the tests and the interference of antibody responses generated by previous infections. Here we investigate the diagnostic characteristics of a newly developed antibody (HA1) protein microarray using data from cross-sectional serological studies carried out before and after the pandemic of 2009. The data are analysed by mixture models, providing a probabilistic classification of sera (susceptible, prior-exposed, recently infected). Estimated sensitivity and specificity for identifying A/2009 infections are low using HI (66% and 51%), and high when using A/2009 microarray data alone or together with A/1918 microarray data (96% and 95%). As a heuristic, a high A/2009 to A/1918 antibody ratio (>1.05) is indicative of recent infection, while a low ratio is indicative of a pre-existing response, even if the A/2009 titer is high. We conclude that highly sensitive and specific classification of individual sera is possible using the protein microarray, thereby enabling precise estimation of age-specific infection attack rates in the population even if sample sizes are small.

## Introduction

Yearly epidemics of influenza A are the cause of a variable burden of disease that can be substantial in years with high influenza activity [1]–[4]. To date, the methods of choice for classification of individuals as infected, immune, or susceptible using serum are the virus neutralization, complement fixation, and hemagglutination inhibition (HI) tests. These tests have a long history, have been validated against positive and negative samples, and have proved their value in countless studies.

Traditionally, the gold standard for detecting influenza infections is by the use of paired serum samples, the first taken in the acute phase of infection and the other several weeks later. A significant (usually fourfold) increase in antibody titers is subsequently taken as evidence for recent infection. In practice, however, it is both costly and logistically challenging to obtain such samples. Consequently, residual or other one-point serological samples are often used instead, and classification is based on a high antibody titer in the one-point sample. Such classifications, however, may lack in sensitivity, especially when it comes to distinguishing between persons that have been infected recently and persons that have been infected with similar viruses in the past.

Moreover, in comparative studies when multiple antigens need to be tested the traditional tests are laborious, and need a significant amount of serum. Recent studies have made increasing use of novel diagnostic assays based on protein microarrays [5]–[8]. Advantages of the protein array are the smaller volumes of blood, the possibility of simultaneous testing of samples against multiple antigens, and potentially the test characteristics.

In the Netherlands, two serological studies had been conducted before and after the H1N1 pandemic of 2009 [9]. In these studies, samples had been analysed with HI to obtain estimates of the age-specific attack rates, by comparison of post- versus pre-pandemic seropositivity. Here, we analyse a subset of these samples with the newly developed protein microarray. Our aims are to explore the diagnostic characteristics of the microarray, and in particular to investigate whether the microarray would enable reliable classification of persons as being recently infected (with A/2009 H1N1), or having a response resulting from infection(s) in previous years.

The data are analysed using mixture models. In contrast to traditional analyses which use a fixed cut-off value to classify each sample into one class (susceptible, immune, recently infected), mixture models estimate the probability that a sample belongs to one of these classes. Hence, mixture models provide a natural way to include uncertainty in the classification procedure, and also enable investigation of optimal cut-off values [9], [10].

## Materials and Methods

### 1. Data

Two age-stratified population based surveys had been conducted in the Netherlands before and after the pandemic of 2009 [9]. Here, we analyse a structured random subset containing 167 and 190 sera from the earlier study (Table S1). The two samples are stratified by age (0–4, 5–9, 10–19, 20–44, 45–64, and 65+ years), as recommended by the Consortium for the Standardization of Influenza Seroepidemiology (consise.tghn.org). Further, children under the age of five are excluded due to the small number of participants [9], and persons receiving pandemic vaccinations and elderly (65+ years) are excluded because of the interference of vaccination with the test results [8]. We also excluded sera from the pre-pandemic survey that had been collected after 12^th^ of October 2009, which marks the onset of sustained transmission in the Netherlands.

The aim of the earlier study was to obtain estimates of age-specific infection attack rates, and sera had been analysed with a hemagglutination inhibition test (HI). Most of the samples in the earlier study tested negative using HI. To prevent a random sample being drawn that contains mostly test negative sera, we stratify the sampling procedure by HI titer. One group contains sera that tested negative, one group contains sera with a low to intermediate standardised HI titer (positive but <40; henceforth called intermediate titer), and one group contains all sera with a intermediate to high standardised HI titer (≥40; henceforth called high titer). This procedure stratifies the population by age, (standardised) HI titer, and survey (pre- versus post-pandemic). Two strata contain no data, as all persons aged 5–9 years tested negative in the pre-pandemic sample. For the remaining 28 groups we have drawn a random subset for analysis (Table S1). The original surveys contained two random samples of the population, and therefore so do the subsets. Our stratification scheme enables weighing of the sera in the subset to represent a random sample from the Dutch population.

The study was approved by the Medical Ethical Testing Committee of Utrecht University (Utrecht, the Netherlands), according to the Declaration of Helsinki (protocol 66-282/E). Written informed consent was given by participants (or next of kin/caregiver in the case of children) for suitably anonymised clinical records to be used in this study.

### 2. Hemagglutinin (HA1) microarray

The subset of sera from the original study was analysed with a microarray as described earlier [5]–[8]. Briefly, recombinant proteins were produced in human embryonic kidney cells (HEK293) and purified by HIS-tag purification (purity more than 95%), as specified by the manufacturer (Immune Technologies, New York, USA). Oncyte avid nitrocellulose film-slides containing 64 pads per slide were used (Grace bio-labs, Bend, USA), and spot signals were quantified by the use of a Scanarray scanner (Perkin Elmer, Waltham, USA) using an adaptive circle quantification method. Finally, conjugates consisted of goat anti-human IgG (Fc-fragment specific) conjugated with Dylight649-fluorescent dye (Jackson Immuno Research, West Grove, PA, USA).

Table 1 shows the antigens included in the study. Notice that next to the antibody response against the A/2009 (H1N1) pandemic virus, we tested the samples against a range of other antigens, among which A/1918 (H1N1). The hemagglutinin of H1N1 virus of 1918 is genetically and antigenically related to the 2009 virus [11]–[14]. Readers of each test (HI and microarray) were blind to results of the other tests, and had no access to ancillary information (age, sex).

**Table 1:** Overview of HA1 antigens included in the protein microarray.

Strain	Subtype
**A/South Carolina/1/1918**	**H1N1**
A/WS/1933	H1N1
A/New Caledonia/20/1999	H1N1
A/Brisbane/59/2007	H1N1
**A/California/06/2009**	**H1N1**
A/Canada/720/2005	H2N2
A/Aichi/2/1968	H3N2
A/Wyoming/2/2003	H3N2
A/Brisbane/10/2007	H3N2
A/Vietnam/1194/2004	H3N2
A/Chicken/Netherlands/1/2003	H5N1
A/Guinea fowl/Hong Kong/WF10/1999	H7N7

Antigens in bold have been used for classification of persons as being susceptible to, immune against, or recently infected with pandemic virus (A/2009 H1N1).

### 3. Mixture model

We use a mixture model to provide a probabilistic classification of individual samples and estimate age-specific infection attack rates. The mixture model contains three component distributions that model the responses across age groups. The first distribution describes samples of low antibody titer, pertaining to susceptible persons. The second distribution describes samples of intermediate antibody titers and aims to identify persons that have pre-existing antibodies, hereafter named the immune component distribution. The third distribution describes samples of high titer, and aims to identify persons infected during the pandemic.

The susceptible and immune component distributions are fitted to pre- and post-pandemic data, while the infected component distribution is fitted to the post-pandemic data only. We assume that there are no age dependencies in the component distributions, and fit Gaussian distributions to the log_2_ antibody titers. We collect the means (*μ*_sus_, *μ*_imm_, and *μ_i_*_nf_) and standard deviations (*σ*_sus_, *σ*_imm_, *σ*_inf_) of the distributions in parameter vectors (**θ**_sus_, **θ**_imm_, **θ**_inf_), and denote by *f*(*x*; **θ**) the densities of the distributions.

The weights of the distributions are determined by two mixing parameters per age group, *viz. q_a_*, the probability that a person in age group with label *a* belongs to the immune component, and *p_a_*, the probability that a person with age label *a* is in the infected component. Hence, *1- q_a_* and *1-p_a_-q_a_* are the probabilities that a person belongs to the susceptible component in the pre- and post-pandemic surveys. Notice that we make the implicit assumption that the fraction of persons in the immune component remained constant in the short time span (≤6 months) between the two surveys. In the following, the age-specific weights are collected in vectors **p** and **q**. At the individual level, the probability that a person in the post-pandemic survey with age label *a* is infected is given by the product of the mixing parameter *p*_a_ and the local density of the infected component distribution, normalised by the sum of these quantities over all component distributions (susceptible, immune, infected).

The statistical analyses are based maximization of the log-likelihood. In the following we denote by *n*_pre_ and *n*_post_ the number of samples in the pre- and post-pandemic survey, by *d*_pre_(*i*) the log_2_ antibody titer of sample *i* in the pre-pandemic study, by *g*(*i*) the age label of sample *i*, and by *w*(*i*) the population weight of sample *i*. With these notational conventions the log-likelihood of the pre-pandemic data can be written as
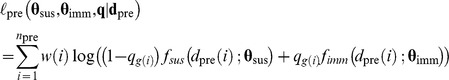
and the log-likelihood of the post-pandemic data is given by



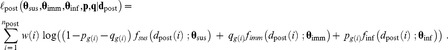



The total log-likelihood is given by the sum of the pre- and post-pandemic log-likelihoods. In practice, the above formulations need to be adapted slightly to account for left-censoring of samples below the detection limit [9]. Notice furthermore that HI measurements are interval-censored, as the data are based on analysis of serial dilutions, and this has been taken into account in the analysis of HI data [9].

To investigate whether classification of individual samples can be improved by the inclusion of a second antigen, we extend the univariate mixture model described above to a bivariate mixture model. The analysis of the extended model runs along the same lines as outlined above, the main difference being that the component distributions are now specified not by a single mean and standard deviation, but by two means (e.g., 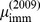 and 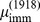), two standard deviations (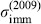 and 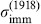), and a covariance (*ρ*_imm_). Hence, the equations remain the same, but in this case the parameter vectors contain five instead of two elements.

### 4. Estimation

The mixture models are fitted using Markov Chain Monte Carlo methods. Specifically, we use a random walk metropolis algorithm with normal proposal distributions and the current value as mean [15]. For each analysis, we run the process for 100,000 cycles, and obtain a thinned sample of 24,000 after a burn-in of 4,000· Convergence and mixing are assessed visually. A maximum likelihood estimate of the parameters is obtained, and limits of 95% parameter confidence intervals are determined by taking 2·5% and 97·5% quantiles. All statistical procedures have been programmed in R version 3.0.0.

## Results

**Figure 1:**
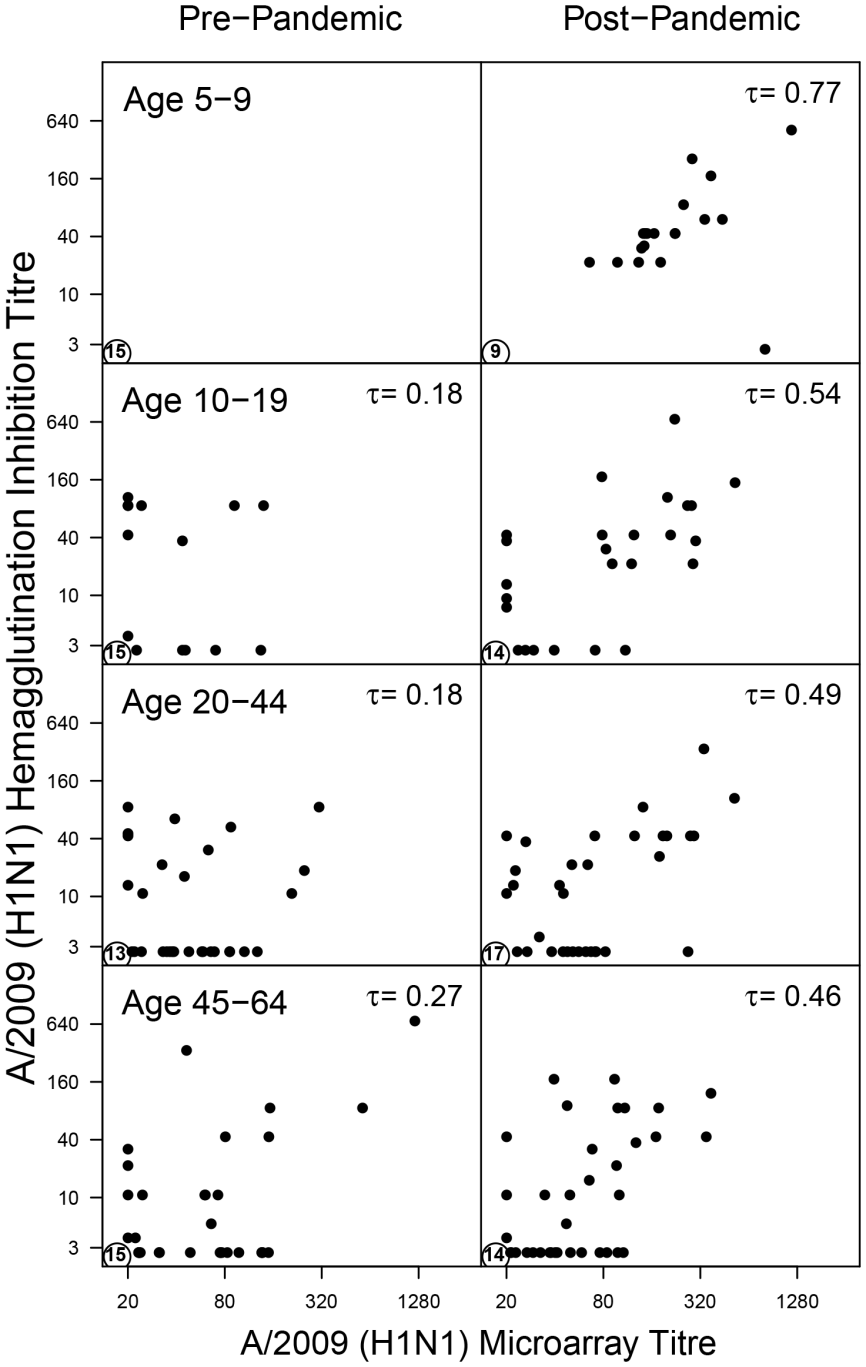
Standardised hemagglutination inhibition titers as a function of A/2009 microarray titers. Data are stratified by study and age group (5–9, 10–19, 20–44, and 45–65 years). The bottom left corner in each panel shows the number of samples that tested negative in both assays. The top right corner shows Kendall's tau, a nonparametric correlation coefficient.

There is a positive overall correlation between HI and the microarray response to A/2009 (Kendall's tau  = 0·45, p-value <0·001). The correlation is stronger in the post-pandemic study (tau  = 0·57, p-value <0·001) than in the pre-pandemic study (tau  = 0·28, p-value <0·001), and is strongest in young children (5-9 years) in the post-pandemic study (tau  = 0·77, p-value <0·001). A further comparison shows that 100 out of 357 samples (28%) test negative in HI but have a positive response in the microarray (Figure 1). The opposite is true for just 23 persons (6%). The number of people that test negative in the HI but positive in the microarray increases with age (p-value <0·001, tested with a logistic regression) and does not appear to be affected by seasonal vaccinations (p-value  = 0·49).

**Figure 2:**
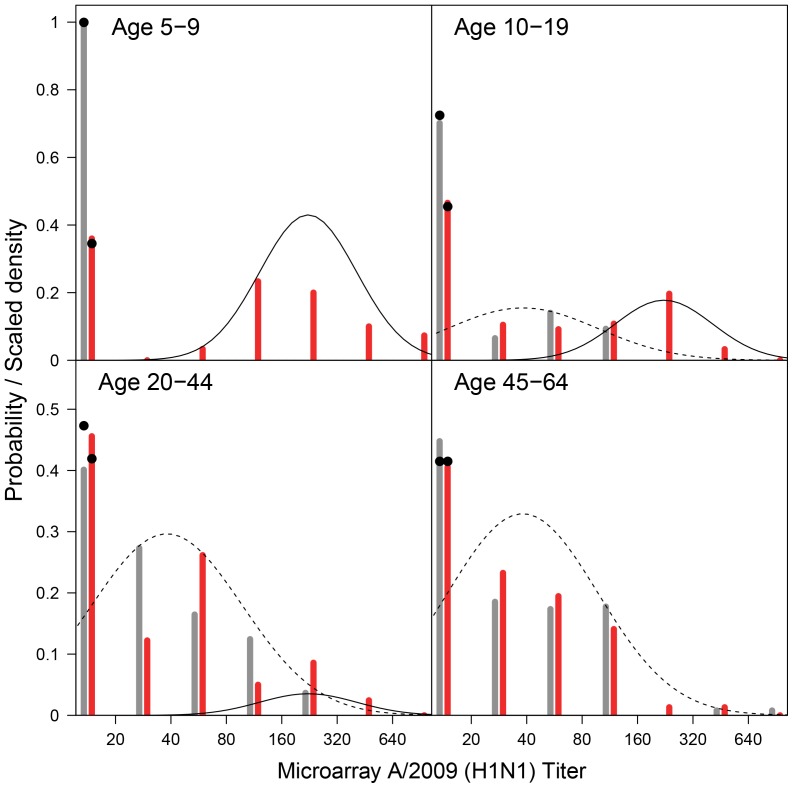
A/2009 (H1N1) microarray titers (bars) and the fitted mixture distributions (lines). The data are aggregated as follows: <20, 20–40, 40–80, 80–160, 160–320, and 320–640. Grey and red bars represent pre- and post-pandemic data, respectively. The solid and dashed line represent the immune and infected component distribution, respectively. The cumulative probabilty density of the mixtures below the detection limit of 20 are marked with black dots.

In young children (5–9 years) there is a perfect distinction between persons that were likely infected, and those that remained susceptible. In fact, in the pre-pandemic study there are no young children with a positive test result in the A/2009 microarray, while 64% of the participants has a titer higher than 65 in the post-pandemic study (Table 2), yielding a clear bimodal distribution of antibody titers in the post-pandemic study (Figure 2). A bimodal distribution is also apparent in older children (10–19 years) and younger adults (20–44 years) in the post-pandemic study, albeit less pronounced. In older adults (45–64 years), the bimodality of the distribution of antibody titers in the post-pandemic study has disappeared.

**Table 2:** Overview of the microarray data, stratified by age, cut-off for seropositivity, and study period (pre- versus post-pandemic).

	A/2009 (H1N1) Microarray Titer	Age Group (years)			
		5–9	10–19	20–44	45–64
Pre-pandemic	>20	0	0·30	0·60	0·55
	>40	0	0·23	0·33	0·37
	>65	0	0·13	0·22	0·30
Post-pandemic	>20	0·64	0·53	0·54	0·59
	>40	0·64	0·43	0·42	0·36
	>65	0·64	0·43	0·25	0·22
	>20	0·64	0·24	−0·05	0·04
Post-Pre	>40	0·64	0·20	0·10	−0·01
	>65	0·64	0·30	0·03	−0·08

For each group the seroprevalence, i.e. the fraction with a titer higher than the cut-off, is shown. Also shown are the seroprevalence differences between the post-and pre-pandemic samples.

Subtracting post- and pre-pandemic prevalences yield rough estimates for the age-specific infection attack rates, suggesting that infection attack rates are high in young children (64%) and low in older adults (<4%)(Table 2). Formal analyses using mixture models yield comparable estimates (Table 3). Above the age of 20, the attack rates decrease less sharply in the bivariate model, as the bivariate model is better able to identify infected persons (see below).

**Table 3:** Age-specific estimated probabilities (weights) of the component distributions.

		Estimated Probability (95% CI)		
Age	Component	Univariate microarray	Bivariate microarray	Univariate HI
5–9	Susceptible (pre)	1·00 (0·79;1·00)a)	1·00 (0·84;1·00)a)	1·00 (0·52;1·00)
	Susceptible (post)	0·35 (0·12;0·61)	0·37 (0·06;0·59)	0·40 (0·01;0·64)
	Immune	0·00 (0·00;0·21)a)	0·00 (0·00;0·16)a)	0·00 (0·00;0·48)
	Infected	0·65 (0·31;0·82)	0·63 (0·36;0·89)	0·60 (0·25;0·82)
10–19	Susceptible (pre)	0·63 (0·37;0·82)	0·44 (0·24;0·66)	0·76 (0·21;0·98)
	Susceptible (post)	0·36 (0·12;0·56)	0·22 (0·03;0·40)	0·56 (0·02;0·72)
	Immune	0·37 (0·18;0·63)	0·56 (0·34;0·76)	0·24 (0·02;0·79)
	Infected	0·27 (0·11;0·49)	0·22 (0·08;0·46)	0·20 (0·11;0·61)
20–44	Susceptible (pre)	0·30 (0·06;0·43)	0·07 (0·04;0·20)	0·89 (0·22;0·97)
	Susceptible (post)	0·25 (0·02;0·36)	0·00 (0·00;0·10)	0·81 (0·09;0·89)
	Immune	0·70 (0·57;0·94)	0·93 (0·80;0·96)	0·11 (0·03;0·78)
	Infected	0·05 (0·00;0·15)	0·07 (0·03;0·15)	0·08 (0·02;0·26)
45–64	Susceptible (pre)	0·22 (0·05;0·4)	0·12 (0·04;0·24)	0·91 (0·17;0·97)
	Susceptible (post)	0·22 (0·02;0·37)	0·07 (0·00;0·18)	0·88 (0·07;0·91)
	Immune	0·78 (0·60;0·95)	0·88 (0·76;0·96)	0·09 (0·03;0·83)
	Infected	0·00 (0·00;0·10)a)	0·05 (0·00;0·14)	0·03 (0·01;0·20)

Three scenarios are considered, viz. a univariate model that uses A/2009 (H1N1) microarray data (‘Univariate microarray’), a bivariate model that uses A/2009 (H1N1) and A/1918 (H1N1) microarray data (‘Bivariate microarray’), and a univariate model of hemagglutination inhibition data using A/2009 (H1N1) (‘Univariate HI’). See Table S2 and Table S3 for estimates of the parameters of the distributions.

a) one-sided confidence interval.

**Figure 3:**
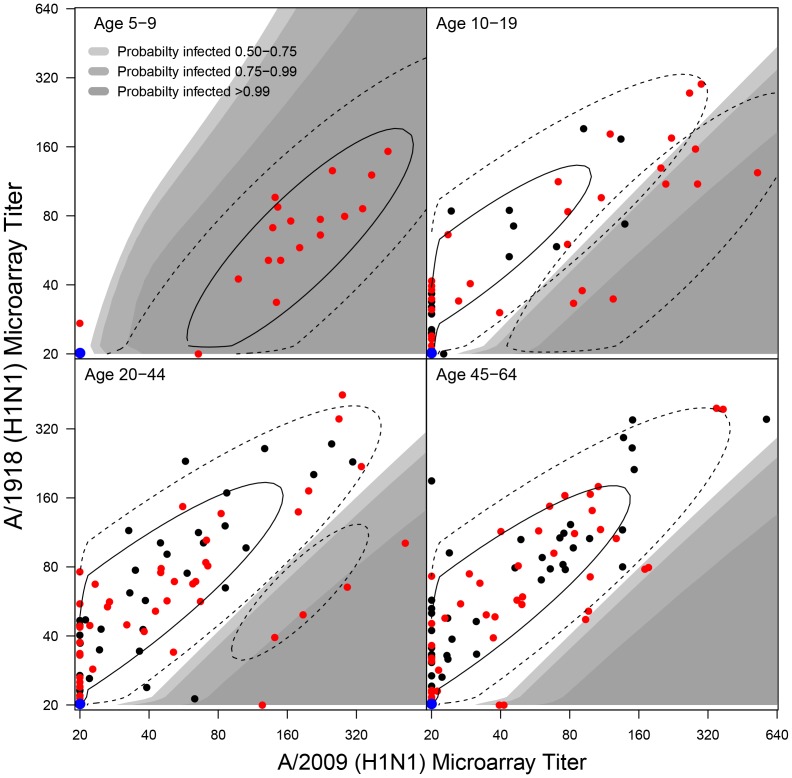
Model fit of bivariate H1N1 microarray data (A/2009 versus A/1918). Black and red dots represent the data, i.e. pre- and post-pandemic samples. Contours indicate the densities of the immune and infected distributions weighted by the the estimated age-specific infection probabilities (Table 3). The blue dots indicate that multiple samples are negative to both A/1918 and A/2009 (age-specific numbers in the pre-pandemic survey: 15, 20, 16, 19; post-pandemic survey: 9, 19, 19, 17). Grey areas indicate the regions with high probabilty that a post-pandemic sample is infected with A/2009. The susceptible component is placed largely placed beneath the detection limit and is not displayed.

Figure 3 shows the bivariate microarray data (dots), the fitted bivariate immune and susceptible component distributions (contours), and the regions of high estimated infection probability (shaded areas). There is a positive correlation between the test results for A/2009 and A/1918, as would be expected. Further, the infected component distribution is located at modestly higher A/2009 titers than the immune component distribution, and the A/2009 antibody titer alone appears to be insufficient to separate infected persons from those with pre-existing responses (the immune component). In fact, the main difference between the infected and immune component distributions is that the former is located below the latter in the A/2009-A/1918 plane. In other words, a person with a certain A/2009 antibody titer likely has some pre-existing immunity if it also has a high A/1918 antibody titer; if it has a low A/1918 titer, it is more likely that the person has been infected by A/2009 virus.

**Figure 4:**
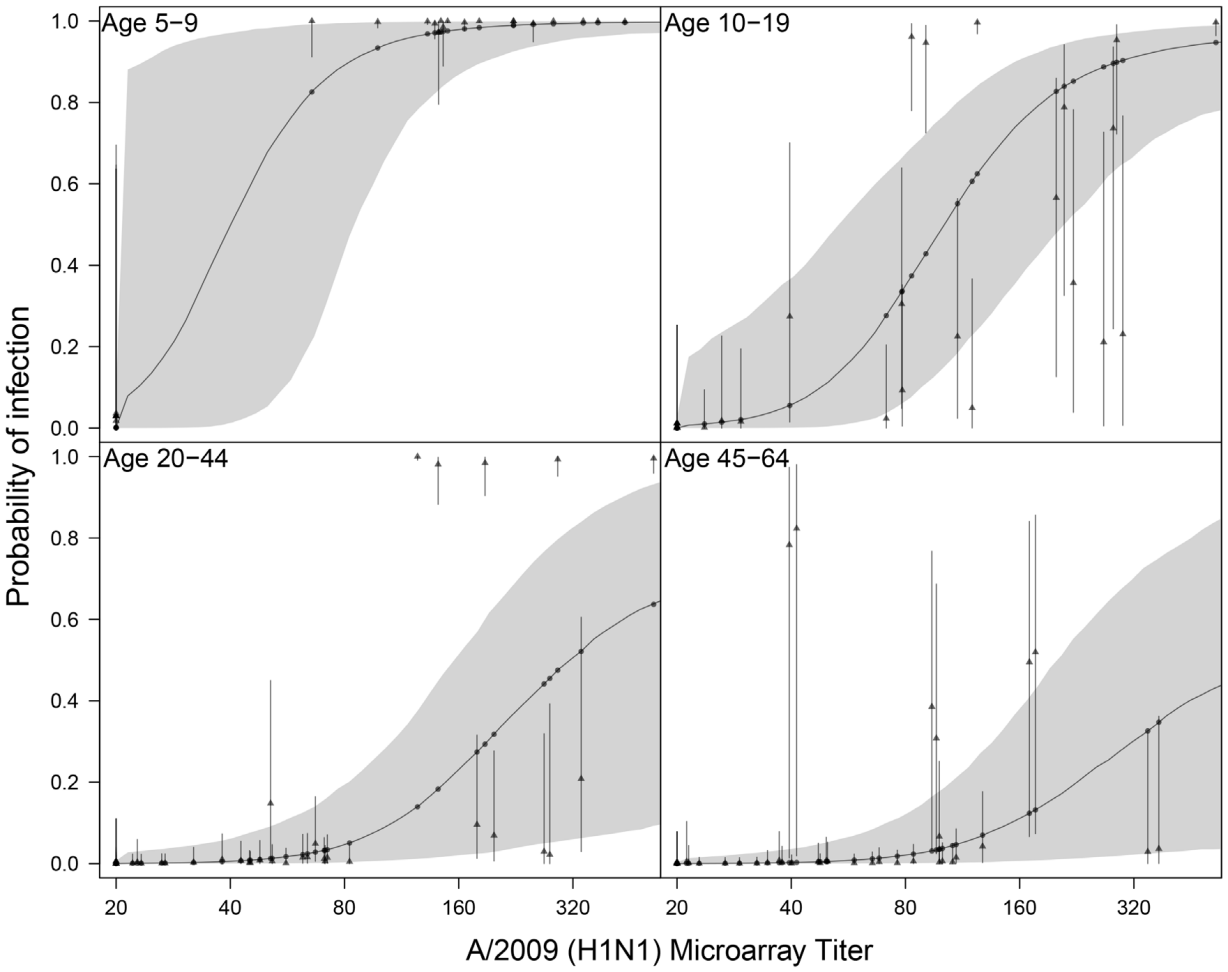
Classification of sera in the uni- and bivariate mixture analyses as a function of the microarray response to A/2009 (H1N1). Shown are the estimated probabilty of infection in the univariate mixture (dots and solid line) with associated 95% confidence envelope (shaded area), and for each sample the corresponding estimates in the bivariate mixture (triangles) with associated 95% confidence intervals (bars).

We further evaluated the diagnostic characteristics of the microarray by analysing classification of post-pandemic sera. In general, classification is most precise in the bivariate microarray (Figure 4). For instance, in young adults (20–44 years) many sera of intermediate to high antibody titers in the A/2009 microarray (160–640 titer) cannot be classified as infected (estimated infection probabilities range from 30–50% with confidence intervals ranging from <10% to >70%). Inclusion of A/1918 in the analysis strongly improves classification; samples with low A/1918 antibody titer have estimated infection probabilities of >95% with small confidence ranges, and samples with high A/1918 scores have estimated infection probabilities under 20% (confidence limits range from 20%–40%).

True infection statuses are unknown in the post-pandemic survey, but we can safely assume that pre-pandemic samples do not belong to persons who have been infected with A/2009. We exploit this fact to investigate how many pre-pandemic samples would be misclassified as infected. Each sample in the pre-pandemic survey has a certain estimated infection probability, and we report the expected number of misclassifications i.e. the infection probabilities cumulated over all positive pre-pandemic samples. The bivariate microarray yields the lowest percentage of misclassifications (8·6 out of 64; 13%), followed by the univariate microarray (18·1 out of 64; 28%), and the HI analysis (16·1 out of 38; 42%).

**Figure 5:**
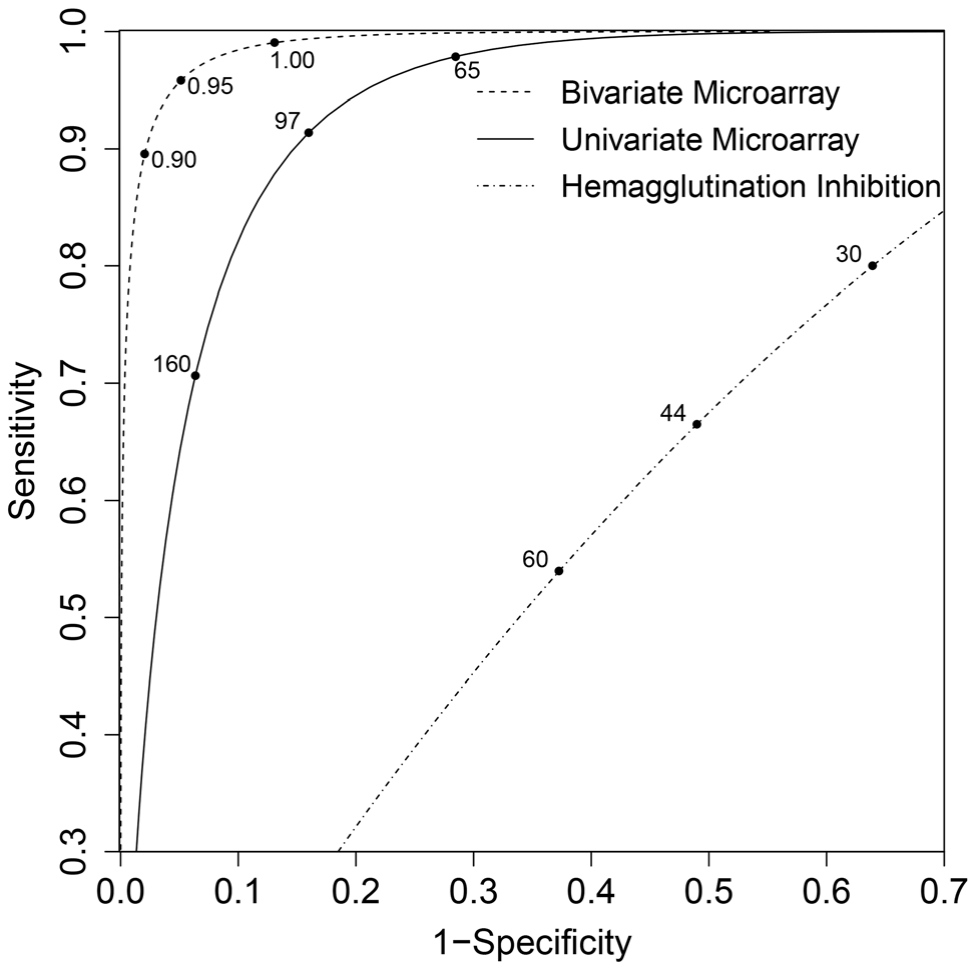
Receiver operating characteristic (ROC) diagram of the univariate model based on HI measurements, the univariate microarray data (A/2009), and the bivariate model of microarray data (A/1918 and A/2009). Maximum sensitivity plus specificity are 66% and 51% for HI (at a cut-off for standardised HI of 44), 91% and 84% for the univariate microarray (at a microarray titer of 97), and 96% and 95% for the bivariate microarray (at a A/1918 to A/2009 ratio of 0·95).

Overall comparison of classifications is investigated in a receiver operating characteristic (ROC) diagram, taking different cut-off values for positive classification (HI and univariate microarray), or taking different values of the A/1918 to A/2009 ratio for positive classification (bivariate microarray) (Figure 5). For HI, maximum sensitivity plus specificity are at a cutoff of 44, with sensitivity and specificity of 66% and 51%. The univariate microarray scores higher with sensitivity and specificity of 91% and 84%, at a microarray titer cutoff of 97. The bivariate mixture scores even higher with sensitivity and specificity of 96% and 95%, at a microarray titer ratio of 0·95 (A/1981 to A/2009).

## Discussion

Using mixture model analyses of two population-based serological studies [9], we have shown that classification of sera for infection with influenza (A/2009 H1N1) is possible using a recently developed protein (HA1) microarray. Sensitivity and specificity are high in the univariate as well as the bivariate model. In the microarray, misclassification of pre-pandemic samples as infected occurs infrequently, and estimates of infection attack rates are comparable to published figures, with comparable precision even though our sample size is much smaller than in earlier studies [9], [16].

Our analyses have uncovered that classification of sera belonging to persons infected with A/2009 (H1N1) works best when using the A/2009 and A/1918 antigens together. The explanation is that in the univariate analysis the component distribution of infected persons has a considerable overlap with the immune component distribution. Incorporation of A/1918 in the analysis reduces the overlap of the two distributions substantially, resulting in classifications that have higher estimated specificity and sensitivity in the bivariate than univariate analysis (Figure 5). When using a more distantly related A/2007 antigen in combination with A/2009 in the bivariate mixture, classification of samples is not improved, the reason being that there is little cross-reactivity between A/2009 and A/2007 antigens (results not shown). Hence, our analyses suggest that the use of additional data works best when using a secondary antigen that is closely related to the focal virus, so that a distinction can be made between the specific responses that are the result of infection, and the correlated but less specific responses that result from earlier infections with other viruses. Whether such combinations of viruses that are antigenically related but not almost identical are available for other subtypes, e.g., H3N2 remains to be investigated.

The microarray measures antibody binding and the observed antibody responses are not necessarily protective. It is known, however, that positive responses in the microarray correlate with protection against infection [5], [6]. Furthermore, the microarray analyses are broadly consistent with the analyses based on HI, with the fraction of persons with pre-existing responses increase strongly with age.

In our analyses the estimated susceptible component is placed largely below the detection limit in the HI and microarray analyses, while the immune component still has substantial density below the detection limit (Figures 2–3). This suggests that it may not always be easy to distinguish susceptible persons from those having been exposed before. One question for future studies is whether classification of persons as being susceptible, immune, or infected can be improved by extending the analyses to more than two antigens, or by using larger datasets.

Throughout, we have assumed that the susceptible, immune, and infected component distributions are independent of age. This is done for simplicity and since allowing for age-dependence in the component distributions would lead to identifiability problems, especially in older adults. As it is, the fit of the infected component distribution is strongly informed by children. However, visual inspection of the locations of the pre- and post-pandemic samples across all age groups in the A/2009-A/1918 plane shows that most lie within the regions of high support of the model, i.e. there are very few outliers. This indicates that the model and the fitted mixture model describes the data well, not only in children but also in older age groups.

## Supporting Information

Table S1 Number of samples in the earlier survey (see main text), and the subset that has been tested with the microarray. Not eligble for selection were pre-pandemic samples collected after October 11 (42 samples), and post-pandemic samples from persons.
(DOCX)

Table S2 Mean and variance of susceptible, immune, and infected component distributions of the univariate mixture model fitted to microarray responses against A/2009 (H1N1), and to the standardised HI titers.
(DOCX)

Table S3 Mean and variance of the susceptible, immune, and infected component distribution of the bivariate mixture fit to A/2009 (H1N1) and A/1918 (H1N1).
(DOCX)
