# Correction: Discrimination of Influenza Infection (A/2009 H1N1) from Prior Exposure by Antibody Protein Microarray Analysis

**DOI:** 10.1371/journal.pone.0118047

**Published:** 2015-02-06

**Authors:** The PLOS ONE Staff

There are errors in Table 1 of the published article. Please see the correct Table 1 below.

**Table 1:** Overview of HA1 antigens included in the protein microarray. Antigens in bold have been used for classification of persons as being susceptible to, immune against, or recently infected with pandemic virus (A/2009 H1N1).

Strain	Subtype
A/South Carolina/1/1918	H1N1
A/WS/1933	H1N1
A/New Caledonia/20/1999	H1N1
A/Brisbane/59/2007	H1N1
A/California/06/2009	H1N1
A/Canada/720/2005	H2N2
A/Aichi/2/1968	H3N2
A/Wyoming/2/2003	H3N2
A/Brisbane/10/2007	H3N2
A/Vietnam/1194/2004	H5N1
A/Chicken/Netherlands/1/2003	H7N7
A/Guinea fowl/Hong Kong/WF10/1999	H9N2
