# Supplementary material for: Discrimination of Influenza Infection (A/2009 H1N1) from Prior Exposure by Antibody Protein Microarray Analysis
Source: PLoS One. 2014 Nov 18;9(11):e113021. doi: 10.1371/journal.pone.0113021 (PMC4236143; doi:10.1371/journal.pone.0113021)
Supplement: Table S1 — Number of samples in the earlier survey (see main text), and the subset that has been tested with the microarray. Not eligble for selection were pre-pandemic samples collected after October 11 (42 samples), and post-pandemic samples from persons. (DOCX) [file pone.0113021.s001.docx]

Table S1. Number of samples in the earlier survey (see main text), and the subset that has been tested with the microarray. Not eligble for selection were pre-pandemic samples collected after October 11 (42 samples), and post-pandemic samples from persons that received a vaccination against pandemic A/2009 (H1N1) (541 samples). Notice that standardised HI titers are presented, and that a standardised HI titer of 2.7 corresponds to an unstandardised HI titer of 5, i.e. a sample that tested negative in the first 1/10 dilution.

| Age |  | Standardised HI Titer | Steens et al. (2011) | Tested |
| --- | --- | --- | --- | --- |
|  | Pre- pandemic | HI ≤2.7 | 19 | 15 |
|  |  | 2.7<HI<40 | 0 | 0 |
| 5-9 |  | HI ≥40 | 0 | 0 |
|  | Post- pandemic | HI ≤2.7 | 12 | 10 |
|  |  | 2.7<HI<40 | 6 | 6 |
|  |  | HI ≥40 | 12 | 12 |
|  | Pre- pandemic | HI ≤2.7 | 27 | 20 |
|  |  | 2.7<HI<40 | 2 | 2 |
| 10-19 |  | HI ≥40 | 7 | 7 |
|  | Post- pandemic | HI ≤2.7 | 32 | 20 |
|  |  | 2.7<HI<40 | 9 | 9 |
|  |  | HI ≥40 | 20 | 10 |
|  | Pre- pandemic | HI ≤2.7 | 69 | 30 |
|  |  | 2.7<HI<40 | 7 | 7 |
| 20-44 |  | HI ≥40 | 6 | 6 |
|  | Post- pandemic | HI ≤2.7 | 106 | 30 |
|  |  | 2.7<HI<40 | 16 | 10 |
|  |  | HI ≥40 | 17 | 10 |
|  | Pre- pandemic | HI ≤2.7 | 101 | 31 |
|  |  | 2.7<HI<40 | 20 | 10 |
| 45-64 |  | HI ≥40 | 6 | 6 |
|  | Post- pandemic | HI ≤2.7 | 218 | 30 |
|  |  | 2.7<HI<40 | 38 | 10 |
|  |  | HI ≥40 | 18 | 10 |
