# Supplementary material for: Discrimination of Influenza Infection (A/2009 H1N1) from Prior Exposure by Antibody Protein Microarray Analysis
Source: PLoS One. 2014 Nov 18;9(11):e113021. doi: 10.1371/journal.pone.0113021 (PMC4236143; doi:10.1371/journal.pone.0113021)
Supplement: Table S2 — Mean and variance of susceptible, immune, and infected component distributions of the univariate mixture model fitted to microarray responses against A/2009 (H1N1), and to the standardised HI titers. (DOCX) [file pone.0113021.s002.docx]

Table S2. Mean and variance of susceptible, immune, and infected component distributions of the univariate mixture model fitted to microarray responses against A/2009 (H1N1), and to the standardised HI titers.

|  | Component | Mean | (95% CI) | Variance | (95% CI) |
| --- | --- | --- | --- | --- | --- |
|  | Susceptible | 1 | (1;9) | 0.71 | (0.04;1.48) |
| A/2009 | Immune | 38 | (25;48) | 0.94 | (0.80;1.20) |
|  | Infected | 223 | (126;310) | 0.61 | (0.49;1.23) |
|  | Susceptible | 4 | (1;5) | 0.49 | (0.02;2.20) |
| HI Titer | Immune | 43 | (1;46) | 1.01 | (0.34;3.81) |
|  | Infected | 66 | (7;122) | 0.93 | (0.93;2.90) |
