# Supplementary material for: Discrimination of Influenza Infection (A/2009 H1N1) from Prior Exposure by Antibody Protein Microarray Analysis
Source: PLoS One. 2014 Nov 18;9(11):e113021. doi: 10.1371/journal.pone.0113021 (PMC4236143; doi:10.1371/journal.pone.0113021)
Supplement: Table S3 — Mean and variance of the susceptible, immune, and infected component distribution of the bivariate mixture fit to A/2009 (H1N1) and A/1918 (H1N1). (DOCX) [file pone.0113021.s003.docx]

Table S3. Mean and variance of the susceptible, immune, and infected component distribution of the bivariate mixture fit to A/2009 (H1N1) and A/1918 (H1N1).

| Component | Parameter | Estimate | (95% CI) |
| --- | --- | --- | --- |
| Susceptible | Mean A/2009 | 9 | (1;11) |
|  | Mean A/1918 | 4 | (1;11) |
|  | Variance A/2009 | 0.16 | (0.02;0.91) |
|  | Variance A/1918 | 2.37 | (0.99;11.4) |
| Immune | Mean A/2009 | 28 | (26;33) |
|  | Mean A/1918 | 50 | (43;56) |
|  | Variance A/2009 | 1.26 | (0.94;1.59) |
|  | Variance A/1918 | 0.77 | (0.6;0.94) |
|  | Covariance | 0.89 | (0.66;1.09) |
| Infected | Mean A/2009 | 182 | (54;270) |
|  | Mean A/1918 | 62 | (26;100) |
|  | Variance A/2009 | 0.73 | (0.38;3.92) |
|  | Variance A/1918 | 0.72 | (0.39;2.99) |
|  | Covariance | 0.62 | (0.22;2.86) |
